# Next Generation Sequencing and Bioinformatics Analysis of Family Genetic Inheritance

**DOI:** 10.3389/fgene.2020.544162

**Published:** 2020-10-23

**Authors:** Aquillah M. Kanzi, James Emmanuel San, Benjamin Chimukangara, Eduan Wilkinson, Maryam Fish, Veron Ramsuran, Tulio de Oliveira

**Affiliations:** Kwazulu-Natal Research and Innovation Sequencing Platform (KRISP), School of Laboratory Medicine and Medical Sciences, College of Health Sciences, University of KwaZulu-Natal, Durban, South Africa

**Keywords:** family genetic inheritance, next generation sequencing, third generation sequencing, genetic variants, phenotypic traits

## Abstract

Mendelian and complex genetic trait diseases continue to burden and affect society both socially and economically. The lack of effective tests has hampered diagnosis thus, the affected lack proper prognosis. Mendelian diseases are caused by genetic mutations in a singular gene while complex trait diseases are caused by the accumulation of mutations in either linked or unlinked genomic regions. Significant advances have been made in identifying novel diseases associated mutations especially with the introduction of next generation and third generation sequencing. Regardless, some diseases are still without diagnosis as most tests rely on SNP genotyping panels developed from population based genetic analyses. Analysis of family genetic inheritance using whole genomes, whole exomes or a panel of genes has been shown to be effective in identifying disease-causing mutations. In this review, we discuss next generation and third generation sequencing platforms, bioinformatic tools and genetic resources commonly used to analyze family based genomic data with a focus on identifying inherited or novel disease-causing mutations. Additionally, we also highlight the analytical, ethical and regulatory challenges associated with analyzing personal genomes which constitute the data used for family genetic inheritance.

## Introduction

Many Mendelian and complex genetic diseases remain unknown despite extensive diagnostic efforts (Shashi et al., 2013). Conventional diagnostic testing methods in most cases return inconclusive results with only less than half of cases receiving a genetic diagnosis (Shashi et al., 2013). Consequently, affected individuals remain without diagnosis and can therefore not be provided with treatment, proper prognosis, beneficial information and appropriate clinical guidance (Stoller et al., 2005). Although Mendelian diseases and complex genetic diseases are individually rare, collectively they affect millions of individuals and families causing negative socioeconomic implications (Angelis et al., 2015; Stoller, 2018). The absence of reliable diagnostic procedures further impedes progress in the development of effective preventative and therapeutic interventions.

Conventional diagnostic testing methods involve clinical assessment followed by laboratory testing. Molecular tests identify candidate gene regions which are subjected to linkage analysis using multiple polymorphic markers within families and individuals that show variation in the trait of interest for positional mapping of the genes (Leal and Speer, 2000). In most cases, large genomic regions containing multiple genes are identified limiting the likelihood of pinpointing the causative genes. Additional information such as phenotype segregation within families or sets of families under examination may be required to narrow down the region of interest and for validation of putative causative genes (Dawn Teare and Barrett, 2005). This approach requires prior understanding of the diseases’ etiology and is therefore only useful whenever such information is available. Other tests such as chromosomal microarray and metabolic testing may be inadequate (Engbers et al., 2008; Miller et al., 2010).

Traditional molecular testing methods greatly relied on Sanger sequencing technology (Sanger et al., 1977). Though efficient for sequencing few short DNA fragments, it is tedious and ineffective when sequencing large sequence fragments. Recent advances in genome sequencing have led to the development of next generation sequencing (NGS) technologies (Morey et al., 2013; Reuter et al., 2015; Heather and Chain, 2016). NGS refers to a collection of technologies that utilize massively parallel sequencing approaches producing millions of short read sequences in a much shorter time, at a much cheaper cost and with higher throughput compared to Sanger sequencing.

NGS-based methods used to analyze genetic variation and their association to particular phenotypes mainly involve case-control study designs with unrelated individuals. These study designs are prone to population stratification bias (PSB) due to genetic differences in ancestry between cases and controls (Freedman et al., 2004). PSB could lead to underrepresentation of *de novo* variants with significant association or overrepresentation of these variations, especially in the absence of association (Thomas and Witte, 2002). Although PSB can be corrected by sampling to enhance homogeneity, false positives could arise even in well-designed studies due to sufficient variation of genetic ancestry (Laird and Lange, 2009). Alternatively, statistical methods could be applied (Price et al., 2006). In cases where variants do not follow Mendel’s law of segregation, family based genetic analyses methods have been used to identify genomic features that do not fall under typical inheritance patterns or to select candidate variants that may be further evaluated (Roach et al., 2010; Wijsman, 2012; Bahlo et al., 2014; Kothiyal et al., 2019).

Family based genetic analysis especially those involving family trios or quartets are crucial for identifying and/or confirmation of rare and common genetic variants (Hansen et al., 2017; Stajkovska et al., 2018; Toptas et al., 2018). In particular, analysis of family trios or quartets provides an effective strategy for the identification of *de novo* mutations that may be linked to disease (Glazov et al., 2011; Jin et al., 2018). Compared to typical variants found in any individual, *de novo* mutations occur at low frequencies and it is quite common that these mutations are overlooked or considered sequencing errors by traditional genetic association analyses strategies (Conrad et al., 2011; Acuna-Hidalgo et al., 2016; Erickson, 2016; Jónsson et al., 2017). Importantly, analysis of family trios or quartets could be used to benchmark variant calling tools in the absence of a reliable “reference” set, aiding sample selection and as a quality control step to improve variant calling and filtering (Bailey-Wilson and Wilson, 2011; Chen et al., 2014; Pilipenko et al., 2014; Teare and Santibanez Koref, 2014; Nutsua et al., 2015; Kómár and Kural, 2018).

The quality of data offered by NGS combined with affordable costs, improved data handling capabilities, increased computational power and efficient bioinformatics analyses tools have immensely facilitated the integration of NGS-based genetic analysis strategies in clinical diagnostics and genetic medicine (Koboldt et al., 2013a; Horton and Lucassen, 2019; Posey, 2019). In this review, we provide an overview of next generation sequencing strategies used for family based genetic analysis to detect genetic variants implicated in Mendelian and rare complex genetic diseases in research and clinical settings. We also discuss the currently available bioinformatics analyses programs and pipelines and considerations that may aid future studies or analytical design.

## NGS Platforms

Currently available NGS platforms apply different approaches to achieve high-throughput sequencing. The differences in sequencing approach in turn influences the sequencing quality, quantity and choice of application. The general approach for a typical NGS run begins with genomic DNA extraction from test samples, library preparation which involves DNA fragmentation, ligation of adaptors, adaptor sequencing, and sample enrichment and finally sequencing (Buermans and den Dunnen, 2014). Several NGS platforms that are currently available (Heather and Chain, 2016; Levy and Myers, 2016).

### Illumina

Illumina^1^, is perhaps the most popular among currently available NGS platforms offering various scalable options that complement requirements of different study designs, cost of sequencing and intended use of the sequencing data (Voelkerding et al., 2009; Buermans and den Dunnen, 2014). These properties present clients with affordable choices and flexibility when designing their studies. Illumina offers a method for selecting an optimum sequencing platform via its sequencing platform comparison tool^2^. The various Platforms produce varying amount of sequencing reads at different sequencing run times (Table 1).

**TABLE 1:** Popular NGS platforms Illumina, IonTorrent, and BGI/MGI.

**Technology**	**Sequencing platform**	**Read length (bp)**	**Data output**	**Run time**	**Recommended application**
Illumina	NovaSeq 6000 System NextSeq 550 System HISeq 3000/4000 System HiSeq X Series	150 PE*	2.4–3.0 Tb 100–200 Gb up to 1.5 Tb 1.6–1.8 Tb	44 h 29 h 4 days 3 days	WGS, WES, PGS
Ion Torrent	Ion GeneStudio S5 System Ion GeneStudio S5 Plus System Ion GeneStudio S5 Prime System Ion PGM 314 System Ion PGM 316 System Ion PGM 318System Ion Proton System (Ion PI Chip)	200 SE 400 SE 200 SE 400 SE 600 SE 200 SE 200 SE 400 SE 200 SE 400 SE 200 SE 400 SE up to 200	10–15 Gb 20–30 Gb 40–50 Gb 30–50 Mb 60–100 Mb 300–600 Mb 600 Mb–1 Gb 600 Mb–1 Gb 1.2–2 Gb up to 15 Gb	19 h 10 h 12 h 2.3 h 3.7 h 3.0 h 4.9 h 4.4 h 7.3 h 2.5 h	WGS, WES, PGS
BGI/MGI	DNBSEQ-T7 DNBSEQ-G400/MGISEQ 2000/BGISEQ 500 DNBSEQ-G400 FAST DNBSEQ-G50/MGISEQ 200/BGISEQ 50	100 PE, 150 PE 400 SE, 100 PE, 150 PE, 200 PE 100 SE, 150 PE 50 SE, 100 SE, 50 PE, PE100	6 Tb 18.75–1,080 Gb 330 Gb 10–150 Gb	24 h ∼78 h 12–13 h 10–64 h	WGS, WES, PGS WGS, WES PGS PGS

The sequencing performance specifications are as per company description. bp, base pair; WGS, whole genome sequencing; WES, whole exome sequencing; PGS, targeted generation sequencing; SE, Single-End reads; PE, Paired-End reads; Gb, Gigabytes; Tb, Terabytes. *Applies to all sequencers.

### Ion Torrent

IonTorrent^3^ sequencing platform provides more or less the same sequencing efficiency in terms of speed and quantity as Illumina. IonTorrent unlike Illumina which uses fluorescent labeling to detect newly synthesized nucleotides uses a semiconductor technology. Detection of newly synthesized nucleotides is based on measuring hydrogen ions released during DNA polymerization using solid state pH meters. Although this method offers shorter sequencing run times compared to Illumina for similar sequence data, there are concerns about the sequencing error rates especially with long sequence homopolymers (Buermans and den Dunnen, 2014; Heather and Chain, 2016; Besser et al., 2018). IonTorrent offers various platforms which support whole genome sequencing (WGS), panel gene sequencing (PGS) and whole exome sequencing (WES) and molecular clinical applications (Table 1).

### Complete Genomics Technology

Complete Genomics technology was developed by Beijing Genomics Institute (BGI) and MGI Tech Co. Ltd. (MGI), a subsidiary of BGI^4^. Complete Genomics technology involves sequencing by ligation, PCR free rolling circle amplification (RCA) and DNA nanoball (DNB) nanoarrays (Goodwin et al., 2016), a process better known as combinatorial probe-anchor synthesis (cPAS) (Fehlmann et al., 2016). NGS sequencing platforms offered by BGI/MGI are adopted for various sequencing applications such as WGS, WES, PGS, transcriptome sequencing, microbial sequencing, epigenetics, and clinical applications (Table 1). In terms of equipment performance for instance, sequencing runtime, sequencing quality and throughput, BGI/MGI sequencers are comparable to other NGS sequencers including Illumina (Fehlmann et al., 2016; Zhu et al., 2018).

### Third Generation Sequencing (3GS): PacBio and Oxford Nanopore

Recently, newer sequencing platforms commonly referred to as Third Generation Sequencing (3GS) have been developed with the aim of sequencing long genomic regions (Reuter et al., 2015; van Dijk et al., 2018). The ultra-long reads produced by these sequencing platforms eliminate the need for the computationally expensive and time-consuming assembly steps of NGS sequencing. Additionally, it allows for identification of structural variants which is not always easy when using short read NGS data.

SMRT (Single Molecule Real Time) sequencing offered by Pacific Bioscience^5^ is able to generate sequence reads of up to 20 Kbs. According to company description, SMRT sequencing has been adopted for applications such as WGS, PGS, RNA sequencing, sequencing of complex populations and for epigenetic studies. Additionally, PacBio have developed a workflow for detecting variants including single nucleotide variants, INDELs and structural variants from the SMRT long read sequences. PacBio RSII is associated with high error rates, however, the new SMRT Sequel II platform is able to generate longer reads at higher throughput and quality at an affordable cost (Table 2).

**TABLE 2:** Long-read sequencing platforms.

**Technology**	**Platform**	**Read length (bp)**	**Data output**	**Run time**	**Recommended applications**
PacBio SMRT	RS II Sequel Sequel II 2.0	∼20 Kb 8–12 Kb ∼15 Kb	up to 1 Gb 3.5–7 Gb 160 Gb/SMRTcell	4 h 30 Min–6 h	WGS, PGS
Oxford NanoPore	Flongle MinION GridION Mk1 PromethION	5–200 Kb^♣^ 2 Mb longest^♣^	1.8 Gb 30 Gb 250 Gb up to 4 Tb	Real time^+^	WGS*, PGS

PacBio SMRT and Oxford NanoPore sequencing platforms currently available. The descriptions provided are from company product specifications. bp, base pair; Kb, kilobase; WGS, whole genome sequencing; PGS, targeted generation sequencing; Gb, Gigabytes; Tb, Terabytes. *Small whole genome sequences. ^♣^All Nanopore sequencers read the entire DNA/RNA fragment presented. ^+^Applies to all Nanopore sequencers.

Oxford Nanopore Technologies (ONT)^6^ is the newest entry in this category offering scalable and portable features that enhance flexibility in terms of laboratory setup. This platform was developed for short to ultra-long read sequencing of DNA/RNA sequences producing high yields especially for large genomes. See reviews by Reuter et al. (2015), Heather and Chain (2016), and Levy and Myers (2016). NGS library preparation is tedious and time consuming. Oxford Nanopore provides a simple, rapid, and library preparation which could be automated thus, does not require extensive training or experience. ONT’s major desirability is the size of the sequencing devices. MinION and FLONGLE for instance are pocket size sequencers thus, enabling mobile genetic testing. The desktop options including GridION and PromethION which produce high throughput sequencing data are easily portable compared to next generation sequencers (Table 2).

Other long read sequencing technologies that are still in early development stages, such as Helicos single molecule sequencing marketed by SeqLL LLC, are yet to achieve effective long read sequencing with efforts still underway. Complete Genome Technology developed by Complete Genomics advances the sequencing by ligation technique used by SOLiD (Supported Oligonucleotide Ligation and Detection) achieving longer sequencing reads and lower error rates in repetitive genomic regions. See review by Ambardar et al. (2016). GnuBIO by BioRad (Hercules, California, United States) is based on microfluidic and emulsion technology. Sequencing is performed on a droplet of DNA effectively simplifying the library preparation step (Klein et al., 2015; Macosko et al., 2015).

There are continued efforts to improve the current state of DNA sequencing mainly to improve the quality, length of DNA sequence, shorten the sequencing procedure and reduce the cost of sequencing. These innovations and discoveries will ease implementation of NGS and 3GS in human genetic research and clinical diagnostic laboratories. In clinical diagnostics, increased sequencing accuracy will guarantee specificity and sensitivity, enabling appropriate disease diagnosis and treatment.

## NGS Strategies for Family Based Genetic Analysis

### Family Based Genome Wide Association Studies

Genome wide association studies (GWAS) is a study method used to detect associations between a genome-wide set of genetic variants and phenotypic traits of individuals within a population, see reviews by Visscher et al. (2012, 2017). Population based GWAS is, however, unable to explain the estimated heritability of the genetic variants detected. To compensate for this limitation, GWAS has been used in combination with linkage analysis to identify both common and rare variants using family based association approach (Benyamin et al., 2009; Ott et al., 2011; Saad and Wijsman, 2014; Ge et al., 2019). For instance, a family based GWAS by Bohman et al. (2017) was able to identify several genes that were implicated in chronic rhinosinusitis with nasal polyps. These genes did not show the genome-wide significant association of 5.0 × 10^–8^. Without linkage analysis these candidate genes could have been overlooked. Using a similar approach Herold et al. (2016) were able to detect with statistical significance novel variants near, or in three genes that influence the onset of Alzheimer’s disease. Mullin et al. (2016) were able to identify two genes associated with bone mineral density using a family based GWAS approach. Using a similar approach, a study by Costantino et al. (2017) was able to identify an association of spondylarthritis with *MAPK14* in a large cohort of multiplex families. Elsewhere, O’Brien et al. (2016) were able to identify novel variants associated with susceptibility to young-onset of breast cancer in a cohort of sisters and their parents.

There are benefits to using family based GWAS (Wijsman, 2012). This approach combines both association and linkage analysis unlike population based GWAS which only provide association analysis (Almlöf et al., 2019). This approach is thus, able to perform genetic analyses that otherwise cannot be conducted on a sample of unrelated individuals. Family based GWAS also offers protection against spurious association due to population substructure and provides robustness against difficulties in genetic interpretation and misspecification of the phenotype model. This approach also allows for identification of genotyping errors and testing whether variants are inherited or *de novo*. These properties make family based GWAS useful in the initial and replication stages of a GWAS study and the selection of appropriate markers (Laird and Lange, 2009).

Family based GWAS is not without disadvantages. Like population based GWAS, it relies on large pedigrees (Herold et al., 2016; Bohman et al., 2017). Recruiting such large numbers of related individuals could be challenging. The large sample size is essential for achieving realistic effective size, but it does come with additional costs and logistical challenges whilst complicating the experimental design. Also, genotypes required for GWAS are normally typed using SNP-chips incorporating hundreds of thousands of SNPs. The genotyping process may introduce errors that may impact genotype calling and classification in addition to its expensive cost. Although, advances have been made to improve data cleaning and genotype calling algorithms, missing, or misclassification errors may not be entirely eliminated. In family based GWAS, these errors can be identified by determining the plausibility of the off-spring’s genotype given the parental genotypes. While it would be logical to exclude misclassified genotypes from the dataset, removing them could result in inflated significance levels (Laird and Lange, 2009). Apart from cleaning genotyping errors, family based GWAS requires an additional step to filter Mendelian inconsistencies where genotypes violating Mendel’s genetic inheritance law are identified and excluded from the dataset. The robustness of family based GWAS is derived from its design which is conditional and almost model free, however, this approach may at times lack associating statistical power comparable to for instance, population based GWAS (Laird and Lange, 2006, 2009).

### Target Specific Sequencing

Target specific sequencing restricts search for genetic variants to the genomic regions of interest. These regions are decided upon based on previous genetic information regarding the disease under investigation. Target specific sequencing approach utilizes gene panels containing a set of genes known to be associated with the disease or phenotype under study. These panels could be purchased with pre-selected content or they could be custom made to contain genomic regions or genes of interest. Advantages of using this approach include low cost of sequencing due to the smaller genomic region considered. The small genomic regions being sequenced allow for higher sequencing depths which enhances detection of rare genetic variants, short insertions and deletions (INDELs), copy number variants (CNVs), alleles occurring at low frequencies and causative or inherited mutations all in a single assay (Lin et al., 2012).

#### Panel Gene Sequencing (PGS)

PGS involves selective enrichment of genes or genomic regions known to be associated with diseases, biological function or pathways as suggested by other genetic analysis. Genomic regions commonly targeted include exons, introns, promoter sequences, or other highly conserved regions of biological significance. Previously, Sanger sequencing technology was used to sequence each of these genomic regions, however, more efficient methods based on NGS platforms have been developed. Currently there are two approaches employed for targeted gene capture including hybridization-based and non-hybridization-based approaches. See review by Lin et al. (2012) for more gene capture methods. This method is attractive for detection of genetic variants associated with monogenic diseases or traits where variants are directly associated and are localized to specific genomic regions (Gulilat et al., 2019).

The design strategy of target specific sequencing allows for detection of causative variants making it well-suited for analysis of family genetic inheritance. A study by Okazaki et al., in 2016 used targeted gene approach to test for Mendelian disorders in 17 families that made up a total of 20 syndromic and non-syndromic patients (Okazaki et al., 2016). In their study, a panel consisting of 4,813 genes associated previously characterized clinical phenotypes were sequenced using the TruSight One panel (Illumina, San Diego, CA). Their analysis was able to positively identify causative variants in approximately 50% of the syndromic patients and approximately 17% for the non-syndromic patients. Overall, in this study the targeted gene sequencing approach using NGS outperformed traditional genetic testing methods such as karyotyping and chromosomal microarray analysis. This study also reported better performance of the targeted gene sequencing approach compared to WES (Okazaki et al., 2016).

PGS is advantageous in that it eliminates superfluous data that could negatively impact the analysis by using a select number of genes linked to or associated with the diseases or traits of interest. This significantly reduces the computational resources required for storage and analysis which is a common problem that is encountered in WGS and WES projects which generate massive amounts of sequence data. WGS and WES also require extensive bioinformatics work that adds to the total cost of sequencing which are not necessary in PGS assays. The cost of WES and WGS has since become affordable, however, it could still be costly when the sequences of more than one individual are required. Despite its advantages, the appealing quality of PGS is also perhaps its biggest limitation in that only variants within the selected genes can be analyzed (Dillon et al., 2018). Targeted gene sequencing assumes that the suspected variants are casual, and the genetic disease is monogenic. As such, this approach is unreliable for genetic diseases that are caused by variants in multiple unlinked genes that may not be included in the targeted gene panel.

#### Whole-Exome Sequencing (WES)

Whole exome sequencing involves the capture and sequencing of all the known protein-coding sequences or exome. In most cases, WES covers approximately 22,000 protein coding genes encoded in the human genome. This approach is also able to capture sequences flanking the coding sequences that may harbor genetic variants (Guo et al., 2012). WES platforms primarily rely on hybridization using oligonucleotide probes to capture the targeted exonic regions for enrichment and library preparation (Keats et al., 2018). Prepared libraries can be sequenced with a NGS platform of choice.

The Illumina HiSeq platform is by far the most popular choice (Chen et al., 2015; Marshall et al., 2015) but the Ion Torrent platform is also frequently used (Franceschi et al., 2017).

The rationale behind the use of WES for genetic analysis of Mendelian and complex diseases is based on the premise that most (over 85%) Mendelian disorders tend to be caused by defects in protein sequences (Kryukov et al., 2007; Stenson et al., 2009; Ng et al., 2010). Additionally, protein coding sequences show higher success rates in identifying variants for monogenic diseases (Antonarakis and Beckmann, 2006). Implementation of WES in clinical genetic diagnosis has had significant success rates (Gorski et al., 2016; Gambin et al., 2017; Mueller et al., 2018). The rate of molecular diagnostic success is seemingly higher when using WES for common disease traits, non-specific phenotypes and rare variants that are non-syndromic compared to other traditional molecular clinical diagnostic tests as shown in studies by e.g., Yang et al. (2013, 2014); Drew et al. (2015), and Long et al. (2015).

WES analysis provides an efficient approach for identifying rare and *de novo* mutations (Zhang, 2014; Posey et al., 2019). For instance, rare genetic variants associated with complex diseases such as schizophrenia have been identified using WES data sets from family trios (Singh et al., 2017). A study conducted by Franceschi et al. (2017) using WES of a family trio with a history of Li–Fraumeni syndrome was able to identify a novel mutation which developed *de novo* in the mother and transmitted to the child. Another study by Chen et al. (2019) reported a *de novo* pathogenic mutation in WES of family trios with epileptic encephalopathy. The efficiency of WES in detecting variants, especially when applied to family trios provides an accurate means to differentiate between sequencing errors and actual biological variation (Bahlo et al., 2014; Retterer et al., 2015; Eldomery et al., 2017; Wright et al., 2018).

### Whole Genome Sequencing (WGS)

WGS is a process by which the entire DNA sequence of any organism is determined. In the case of humans, this includes the chromosomal DNA and mitochondrial DNA. Previously, due to unaffordable costs, NGS was limited to panel-based SNP arrays and targeted gene sequencing approaches. However, current affordable WGS costs (less than $1,000 per genome in the Illumina NovaSeq or BGI/MGI platforms), have incentivized the use of WGS in genetic research and more recently in clinical genetic diagnosis (Fang et al., 2017; Posey, 2019; Rexach et al., 2019).

According to the latest release of the human reference genome (GRCh38), the complete set of protein-coding sequence or exome only constitutes approximately 3.09% (over 90 million nucleotides) of the genome. Although, most Mendelian diseases are caused by deleterious mutations found within the exome (Ng et al., 2010), genetic variations occurring outside the exome sequences that could have significant genetic implications have been identified (Guo et al., 2012). Sequences other than exons include untranslated intergenic regions and introns which have been suggested to alter the regulation of gene expression thereby affecting observed phenotypes.

Analysis of WGS data increases the likelihood of identifying novel variants residing in genomic regions that are not commonly targeted by panel-based and targeted gene sequencing approaches. When applied to families, WGS provides qualitatively unique data compared to that obtained from multiple unrelated individuals. This approach enhances identification of sequencing errors and comprehensive mapping of inheritance states, thus enabling the detection of genomic features showing Mendelian inconsistencies such as copy number variations, and hemizygous deletions (Roach et al., 2010; Kothiyal et al., 2019). For instance, a study using WES data was unable to detect a mutation causing IMAGe syndrome in an imprinted gene (Hamajima et al., 2013). However, using WGS data from a family trio, an IMAGe syndrome causing mutation was identified in an imprinted gene in the proband, thus providing a diagnosis of IMAGe syndrome (Bodian et al., 2014). Imprinted genes do not follow Mendelian inheritance laws, and therefore may be missed especially when methods used are reliant on these laws.

Using WGS in family genetic analysis provides the power to differentiate between sequencing errors and actual mutations. This has been illustrated in a genetic study of a family quartet where candidate genes causing Miller syndrome and primary ciliary dyskinesia in both offspring were precisely identified (Roach et al., 2010). Using WGS, it is also possible to not only identify variants caused by SNPs but also those caused by DNA deletions and insertions (INDELs), structural variants (SVs), and copy number variants (CNVs). Additionally, it is possible to reconstruct the recombination events leading to these variations as shown by Fang et al. (2017) in a study of Prader–Willi Syndrome.

## Linkage Analysis in the Era of NGS

Before NGS, the analysis of Mendelian diseases and other non-disease inheritable traits was achieved using linkage analysis. See Bailey-Wilson and Wilson (2011), for detailed review of linkage analysis in the era of NGS. Linkage analyses aim to find genomic loci containing more than the expected number of co-segregating alleles among affected family members. The assumption here is that, therein, lies the linked genomic loci or genes responsible for the disease in question. This characteristic makes linkage analysis an effective method for identifying rare high-risk disease alleles, however, it is less effective in identifying alleles conferring moderate risk for disease compared to methods such as GWAS. See review (Carlson et al., 2004).

In the advent of NGS, the application of linkage analysis for the identification of disease-causing alleles has been overtaken by methods such as GWAS, PGS, and WGS. However, it is not uncommon for NGS based studies on Mendelian and complex genetic diseases to complement their analysis with linkage analysis. For instance, a genome wide linkage analysis involving 972 bipolar pedigrees was able to locate with significance a genomic region with variants linked with the disease (Badner et al., 2012). Linkage analysis has been used in combination with WES (e.g., in another study of familial goiter) to inform selection of candidate genes for exome sequencing (Yan et al., 2013) and to identify novel candidate genes for familial colorectal cancer (Toma et al., 2019). Combining linkage analysis with NGS based methods provides the ability to differentiate between novel variants and sequencing artifacts or analytical errors in studies involving multiple unrelated individuals, however, rare variants are expected to co-segregate within a family (Bailey-Wilson and Wilson, 2011).

## Bioinformatics Pipelines for Variant Calling and Analyses

### General Variant Calling Workflow Using WES and WGS Data

**FIGURE 1:**
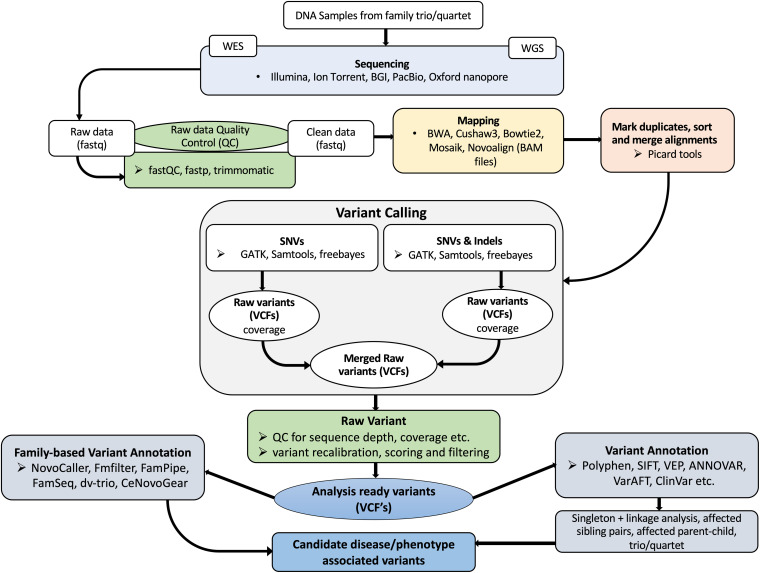
A typical NGS based variant analysis workflow. DNA samples are obtained after clinical, physical assessments and initial molecular diagnosis. WES is preferred for mendelian-type diseases and WGS for unknown or suspected *de novo* variants. Before performing variant analysis, NGS data is pre-processed to remove poor quality sequences. Thereafter, alignment of sequence reads to a reference genome or chromosome, alignment sorting, duplicate removal, variant calling and annotation is performed.

When searching for single nucleotide variants (SNVs) or INDELs in sequence data, different tools are used at various intermediate steps. A typical workflow is to sequence the whole genome or exome, perform quality control and trim, align to a high-quality reference, identify SNVs or short INDELs, and finally to annotate the variants (Figure 1). The GATK^7^ best practices workflows could serve as a guide when setting up variant analysis pipelines.

In the first step, WGS and WES data is subjected to a quality assessment step to remove contaminants such as adapter sequences and poor quality sequences. FastQC is a tool is a tool used to perform quality checks on NGS data providing modular analyses including pre-base analysis of sequencing reads aimed at identifying sequencing problems that may affect downstream analyses (Andrews, 2017). Based on FastQC output, programs such as Trimmomatic (Bolger et al., 2014) can be used to trim adapter sequences and poor-quality reads. These programs are run independently which could affect the outcome of the quality control assessment. For uniformity and reproducibility, fastp (Chen et al., 2018) combines quality control, adapter trimming, and quality filtering in a workflow that is run once.

The second step involves read alignment of the WGS or WES data to the reference genome. It is advisable to use the latest version of the human genome assembly from the 1000 Genomes project. The process of aligning the raw reads to the reference genome is the most important and often contentious step in the entire workflow. Several tools have been developed to facilitate this crucial step. Among the popular read aligners include BWA (MEM and sample) (Li and Durbin, 2009), Bowtie2 (Langmead and Salzberg, 2012), CUSHAW3 (Liu et al., 2014), MOSAIK (Lee et al., 2014), and Novoalign^8^. While these aligners will generate comparable alignments, MOSAIK’s major attraction is that it can align sequence reads from all major sequencing platforms. In terms of computational efficiency CUSHAW3 outperforms BWA-MEM and Bowtie2. Novoalign is computationally efficient, however, it is a commercial product that can be used with Illumina, Ion Torrent and 454 sequencing platforms.

The third step that follows after performing sequence alignment is variant calling. Variant calling involves comparing aligned reads to the reference and identifying nucleotide variations and INDELS. Popular variant callers such as Genome Analysis Tool Kit HaplotypeCaller (GATK-HC) (McKenna et al., 2010), Samtools mpileup (Li et al., 2009), Freebayes (Garrison and Marth, 2012), SNPSVM (O’Fallon et al., 2013), RTG (non-commercial version 3.9.1)^9^, DeepVariant (Poplin et al., 2018), varScan (Koboldt et al., 2013b), and Torrent Variant Caller (TVC) (Life Technologies, Rockville, MD), are widely used in genomic variant analyses. VarScan has recently been extended to also identify variants from Samtools mpileup output BAMs (Koboldt et al., 2013b).

In most cases, any aligner can be used together with any of the variant callers. However, low concordance has been reported among the different combinations of the aligners and variant callers as they are influenced by a number of factors including; (1) The sequencing platform used to sequence the data. For example, only Tmap and TVC can be used on Ion Proton data. They also cannot be used for data generated from other sequencing platforms. (2) Quality of the dataset can affect the rate of precision and recall rates of the pipeline. (3) The type of variant of interest, whether SNP or INDEL. Assessment of outputs from different aligner-caller pairs shows that performance can vary based on the type of variant. INDELs are particularly more difficult to call. (4) GC content of the genomic region (Liang et al., 2019). For example, GATK can detect SNPs in the low GC-content region with a relatively low error rate while RTG and VarScan are more suitable for detecting SNPs in high GC-content region when calling *de novo* SNPs. Detailed explanation of these factors has been documented in other reviews (Reinert et al., 2015; Mielczarek and Szyda, 2016). A growing trend is to use the methods ensemble and produce a consensus call set that contains variants called by the most methods.

To simplify the process of variant calling, several automated workflows have been developed that combine different aligners and variant calling tools, coupled with further up and down stream tools to form a complete end to end solution. Some of these pipelines have been compared thus, aiding the selection process for a suitable variant calling pipeline (Hwang et al., 2015). Here we review the relevant freely available alternatives. To facilitate the choice of a pipeline, ToTem (Tom et al., 2018), a tool for automated pipeline optimization has thus been developed. It can be used to test whole pipelines from raw reads or focussing only on the final variant filtering phases. SeqMule (Guo et al., 2015) is an automated variant calling platform designed to overcome the problem of low concordance across variant calling tools. It integrates five alignment tools i.e., BWA (including BWA-backtrack and BWA-MEM), Bowtie, Bowtie2, SOAP2, SNAP, and five variant calling algorithms i.e., GATK (including GATKLite and version 3), SAMtools, VarScan 2, Freebayes, SOAPsnp, and allows various combinations of them via modifying a text-based human-readable configuration file. The intersection of sets of variants from different combinations of tools is used to achieve higher accuracy. Consensus Variant Calling System (CoVaCS) is an automated, highly accurate system with a web-based graphical interface for genotyping and variant annotation of NGS data. It is able to analyze WGS, WES, and PGS data, performing all steps from quality trimming of sequences to variant annotation and visualization. It implements VarScan, GATK, and Freebayes, with a final call set as the consensus call among tools (Chiara et al., 2018).

Some pipelines integrate many variant calling tools for increased sensitivity. Appreci8 (Sandmann et al., 2018) is an automated variant calling pipeline integrating eight different tools to perform valid variant calling. It can be used for calling single nucleotide variants or short INDELs. It works based on a novel artifact-and polymorphism score. BAYSIC (BAYeSian Integrated Caller) is a variant caller that summarizes SNP variant calls produced by different programs. BAYSIC differs from other consensus-based methods in that it calculates independent false positive and false negative error rates for each input method. The user is able to define cut-off values for the tolerable error rates by supplying a suitable posterior probability threshold thus, controlling specificity and sensitivity (Cantarel et al., 2014). Consensus-based methods are effective in reducing error rates, however, it has been shown that some of these tools require normalization. To ensure uniformity *vt normalize* a tool that normalizes all VCF entries to ensure that they are unambiguous and concisely represented (Tan et al., 2015) could be used.

The process of variant calling can be difficult especially when there are conflicting results from different calling tools. This process becomes even more complicated when there is a high rate of false positives and false negatives. Programs such as geck (Kómár and Kural, 2018) compare differential precision of variant calls from two different tools thereby assisting in determination of variant calls.

### Specialized Pipelines for Family Based Variant Analysis

Some variant callers are designed for analysis of NGS from family members. For instance, novoCaller is a read level variant caller that can be used to identify SNPs from pedigree or population based NGS data. This method has been widely used in studies of family trios (Mohanty et al., 2018). FMFilter is an easy to use inheritance model-based tool for analyzing variants from NGS data generated for the analysis of Mendelian diseases (Akgün et al., 2016). It has been developed to work with family based NGS data and requires minimal bioinformatics experience and computational resources to run. FamPipe (Chung et al., 2016) is an automatic analysis pipeline for analyzing sequencing data in families for disease studies. It includes several family specific analysis modules, including the identification of shared chromosomal regions among affected family members, prioritizing variants assuming a disease model, imputation of untyped variants, and linkage and association tests (Chung et al., 2016). FamSeq incorporates family information from the Mendelian genetic model into variant calling process (Peng et al., 2014). dv-trio (Ip et al., 2020) incorporates family trio information from the Mendelian genetic model into variant calling. This program is based on DeepVariant (Poplin et al., 2018) variant caller that uses a deep neural network to call genetic variants. DeNovoGear is a *de novo* variant calling software that analyses somatic and familial sequencing data. The program uses likelihood-based models to filter out false positives and fragment information predict the parental origin of identified variants. The choice of a family based variant caller could be based on the experimental design, computational efficiency and quality of output.

## Genetic Resources for Variant Analysis

The first fully annotated genome was generated by The Human Genome Project^10^. Afterward, the HapMap Project (International HapMap Consortium et al., 2007) produced a haplotype map of the human genome. Currently, The genome build by The 1000 Genomes Project (The 1000 Genomes Project Consortium et al., 2015) provides information on common human genetic variation with significant implications for common genetic diseases and genetic maps of locations of disease causing variants. The International Genome Sample Resource (IGSR)^11^ maintains the 1000 Genomes Project data which is currently the standard reference genome ensuring regular updates and free access.

While the 1000 Genome Project samples across populations, it may not represent some populations. The Genome Aggregation Database or gnomAD^12^ contains summarized exome and genome sequencing data retrieved from a variety of large-scale sequencing projects. The datasets in this database include SNPs and SVs generated from whole genomes and exome sequences from unrelated individuals as part of disease-specific and population genetic studies. gnomAD provides summary data suitable for diagnosis of disease causing genetic variants. The UK biobank samples over 500,000 volunteer participants for genotyping. The genetic data available from this database include high quality genotype calls, extensive information on the SNPs, population structure and imputed data. Information regarding specific genomic loci is provided through an integrated database^13^. This is particularly useful when analyzing variants suspected of causing Mendelian diseases. The information in this database is freely available to researchers and clinicians.

The National Center for Biotechnology Information (NCBI) supports a wide range of genome analyses through various databases. These include The Database of Genotypes and Phenotypes (dbGaP) an archive of studies investigating the interaction between genotype and phenotype (Mailman et al., 2007), the Database of Genomic Structural Variation (dbVar) an archive of human genomic variations including insertions, deletions, translocations and inversions (Church et al., 2010), and the Database of Short Genetic Variations (dbSNP) an archive of SNPs and other variants with detailed information regarding population frequency, genotype data, and mapping information clinical implications (Sherry et al., 2001). ClinVar^14^ is a database of interpretations of clinical significance for human variants. ClinVar uses Human Genome Variation Society (HGVS) nomenclature and MedGen identifiers for genetic conditions (Landrum et al., 2016). MedGen^15^ database provides information about conditions and phenotypes related to medical genetics. Search results are linked to relevant databases where the primary data can be found.

Genotyping using SNPs has been crucial in determining variants associated with disease. Databases such as GWAS Catalog^16^ and GWASdb^17^ are archives of GWAS data. GWAS Catalog extracts traits, SNP-trait associations and sample metadata from published GWAS studies. The database is searchable, visualisable and can be downloaded for integration into other resources. GWASdb archives and curates traits/disease associated SNPs, their functional annotations and disease classifications collected from current GWAS studies. GWASdb provides an interactive interface to facilitate research and help clinicians to fully exploit available GWAS data. The SNP data from these two databases could be used to design related studies and for analyzing genotyping data. Other helpful genome variation resources include the European Variation Archive^18^ and the Human Variome Project^19^ that archive curated information on all types of genetic variation and their associated effects from all species and in human genomes, respectively.

Most of the databases discussed above primarily archive genes and variants. While they provide detailed and annotations and effects on human health, they may not provide clinically tailored information. Disease specific databases such as ClinGen or The Clinical Genome Resource^20^ provides comprehensive information on the relationship between genes and human health with defined clinical relevance. The database is equipped with tools that enable efficient acquisition of actionable disease information. Similar databases include DisGeNET^21^ a large collection of genes and variants associated with human diseases, The Monarch Initiative^22^ that enables phenotype to genotypes analysis by a semantics based approach, eDGAR^23^ a database of gene and disease relationships, MalaCards^24^ a searchable database of human diseases linked to the GenCards–Human Gene Database, Orphanet^25^ an encyclopedia of rare diseases and associated genes, and Geno2MP^26^ a browser that enables users to link genotypes to mendelian phenotypes.

Locus-specific databases (LSDBs) archive collections of curated sequence variants in genes associated with disease. The Online Mendelian Inheritance in Man or OMIM^27^ is a catalog of human genes and associated diseases. The database has a collection for all known Mendelian diseases and over 15 000 comprehensively annotated genes. LSDBs like OMIM are crucial for interpretation and classification genetic variation in research and clinical diagnostic results. Due to the numerous number of LSDBs, The Locus Specific Database list^28^ is a searchable database that eases search for LSDBs for specific diseases.

## Classifying Genetic Variants

Once variants have been identified, an important next step is to annotate each variant according to its genomic location, predict its functional effect on a gene and prioritize those that are beneficial or deleterious (filtering). Variants can generally be classified as neutral, beneficial, deleterious/harmful, or as frameshift. Neutral variants include synonymous variants and these neither harm nor help, beneficial mutations provide an advantage such as conferring protection against disease while deleterious mutations are harmful and may increase the likelihood of conditions such as cancer. Beneficial/harmful mutations also referred to as non-synonymous alter the function of proteins. Frameshift mutations, results from a deletion or insertion of a nucleotide altering every subsequent codon.

Scoring of variants is necessary in order to identify the harmful subset (Eilbeck et al., 2017). Tools for scoring deleterious mutations include Polyphen—A web-based tool to predict the impact of amino acid substitutions on the structure and function of a human protein (Adzhubei et al., 2013) and SIFT (sorting intolerant from tolerant) also a web server designed to predict whether an amino acid substitution is deleterious (Sim et al., 2012). A newer version, SIFT 4G, which is much faster and enables computations on reference genomes is also available (Vaser et al., 2016). Other tools include SnpEff (Cingolani et al., 2012), Variant Effect Predictor (VEP) (McLaren et al., 2016) and SeqAnt (Shetty et al., 2010). Tools such as ANNOVAR (Wang et al., 2010), Variant Annotation and Filter Tool (VarAFT) are able to predict and annotate variants and incorporating information related to Mendelian diseases. ClinVar could be used for identification of medically important variants and associated phenotypes (Landrum et al., 2016). ClinVar output is interlinked with dbSNP (Sherry et al., 2001) and dbVar (Landrum et al., 2014) and MedGen (Louden, 2020) databases. Annotations generated could be viewed using genome browsers such as the ENSEMBL^29^ and UCSC Genome Browser^30^. These browsers provide links to databases such as OMIM, ClinGen, and ClinVar among others for further functional analysis.

In order to increase prediction accuracy, it is recommended to use more than one of the tools above and compare the results. Predictions where two or more tools are in agreement confer more confidence. An even better approach is to use a tool such as Combined Annotation–Dependent Depletion (CADD) (Rentzsch et al., 2018) which objectively integrates many diverse annotations into a single measure (C score) for each variant. CADD scores help interpret the genomes of patients with Mendelian diseases caused by high-penetrance mutations and also prioritize low-penetrance variants found in genome-wide association studies. Furthermore, CADD accurately predicts variants in non-coding regions. A substantial number of SNVs with high CADD scores in noncoding variants have been observed, supporting the hypothesis that mutations in regulatory regions contribute to many diseases. CADD is regularly updated implying that the scores keep improving as more annotations are made available. Variant annotation, prediction and prioritization facilitates the application of variants analysis results to clinical practice for diagnosis, prediction and treatment.

Interpretation of variant functional predictions and annotations could be complicated depending the level of individual capacity. As such, the American College of Medical Genetics and Genomics (ACMG) and the Association for Molecular Pathology (AMP) have published standards and guidelines for the interpretation of sequence variants (Richards et al., 2015). Using these guidelines, Clinical Genome Resource (ClinGen) Pathogenicity Calculator, a configurable system and web service for the assessment of pathogenicity of Mendelian germline sequence variants was developed (Rivera-Muñoz et al., 2018) to support clinical and research investigations. ClinVar terms such as pathogenic, protective, risk factor could be used to describe variants that could be considered for further characterization (Kalia et al., 2017). The use of NGS for clinical application is still growing and is bound to experience challenges (Jamuar and Tan, 2015).

## Cloud-Based Bioinformatics Services for Analysis Genomic Data

Analyzing massive genomic data may require advanced computational resources which may be expensive to acquire and manage. Additionally, researchers and clinicians may not have the computing and/or bioinformatics capacity to organize the various computational tools available into workable pipelines for their analysis. High-performance computing (HPC) environments that require advanced computational platforms are commonly used for NGS projects. While HPC’s may be effective for computational analysis, the issue of limited storage space or computational power are common. Cloud computing could provide a solution to this challenges by offering on demand availability of computer systems resources including storage and computing power over the Internet. The application of cloud computing for bioinformatics and genomics analysis have been reviewed (Zhou et al., 2013; Langmead and Nellore, 2018; Navale and Bourne, 2018). Cloud-based genomic analysis platforms such as Terra^31^ and Seven Bridges are^32^ have been developed to accelerate biomedical research including NGS analysis. A list of available open-source and commercial cloud-based NGS tools have been have been described by Bani Baker et al. (2020).

## Selecting an NGS and Bioinformatics Strategy

**FIGURE 2:**
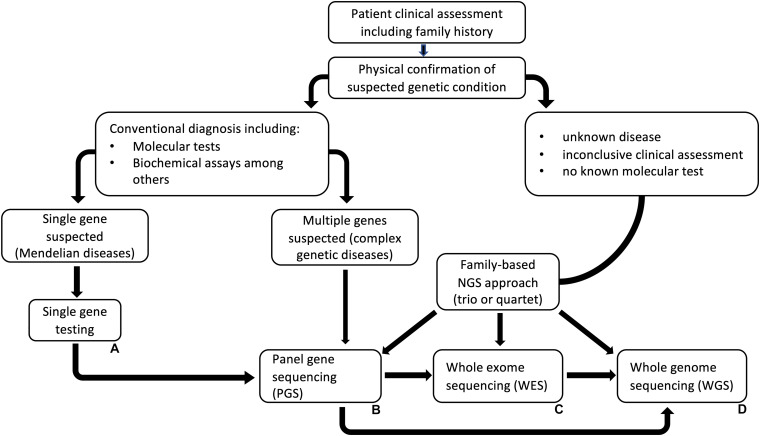
A general next-generation sequencing (NGS) based genetic testing workflow. This is a general guideline for choosing an NGS strategy for analyzing genetic diseases that do not have any known molecular test. **(A)** Single gene testing is suitable when the clinical presentations fit a known disease. If the test is inconclusive, PGS, WES or WGS could be the next approach. **(B)** Panel gene sequencing (PGS), could be used where multiple genes are suspected while **(C)** (WES) and **(D)** (WGS) could be implemented if clinical assessments are inconclusive and in cases where there are no known genetic tests. The illustrated workflow was modified from Shashi et al. (2014).

The choice in strategy for NGS largely depends on the type of genetic disease in the case of clinical diagnosis, or the question to be answered within a research setting (Figure 2). In clinical genetics, sequencing and analysis methods should be well-validated to produce accurate and consistent data that can be reliably used to make clinical decisions. This is very critical considering the psychological, economic, and social implications such information will have on people if and when a hereditary disorder is detected or not. Therefore, appropriate and validated methods spanning from the pre-analytical to the post-analytical phases are crucial in such instances (Zook et al., 2014; Gargis et al., 2015; Highnam et al., 2015). Moreover, prior understanding of the methods for analyzing molecular data is an important consideration in deciding the choice of NGS method. For instance, different NGS methods generate varying sizes of sequencing data, as well as variations in sequence data output [i.e., FASTQ, FAST5, binary base call (BCL)], that require specific methods for analysis. Making these prior considerations helps to save time and money by streamlining the processes and by producing data that meets the requirements for clinical diagnosis.

Different strategies can also be considered depending on whether sequencing is being done for known diseases, multi-gene diseases, or unknown diseases. When a particular disease is known, as well as its clinical features and association with a specific gene, single-gene testing is a more appropriate approach. This approach has an advantage of focusing only on a single gene which results in the known phenotype. However, specialized clinical expertise is paramount with such an approach (Xue et al., 2015). For multi-gene diseases, gene panels are a more feasible strategy for sequencing. Instead of testing one gene at a time, NGS gene panels simplify sequencing and add analytical sensitivity to the diagnostic test. With this strategy, all possible genes associated with a clinical outcome can be targeted at once, or a conservative approach can be used to target only specific genes strongly associated with a disorder. Whichever the strategy used, it is important to understand that some genes which are linked with a disease based exclusively on association studies, do not always result in the expected phenotype. A more pragmatic approach would be to first choose genes that are certainly associated with disease, then genes associated with disorders that have overlapping phenotypes with those of the primary disorders (i.e., for differential diagnosis), and then choose whether to include genes for certain phenotypes associated with syndromic and non-syndromic forms (Xue et al., 2015). For unknown diseases, whole exome sequencing (WES) is the most reasonable strategy to diagnostic testing. WES does not need hypothesis-driven targeted approaches of sequencing specific genes. However, the interpretation of results is better informed when complemented with a thorough history and background information on the phenotype (Xue et al., 2015). Ultimately, the strategy used could aid as a screening tool and has to be sensible enough to provide accurate clinical diagnosis.

The size of the gene, sequence quality, number of reads, and depth of coverage required should be used to direct the choice of the NGS application used. For instance, WGS can help to identify genetic variants which affect phenotypes that are transmissible from parent to offspring. However, it is expensive to produce WGS to a depth that is sufficient to find variants that affect phenotypic expression (Warr et al., 2015). In such cases, target specific sequencing such as WES would be preferable, as the human exome is only ∼3% of the genome, exons average <200 bp in length, and WES only focuses on the coding region of the genome (Meienberg et al., 2015). This allows for sequencing of only the relevant regions without incurring the cost of sequencing the entire genome.

When sequencing larger genome sizes and or in *de novo* sequencing, long read sequencing becomes preferential, as short reads tend to be more challenging to reconstruct, especially around homologous and repetitive sequence regions (Mantere et al., 2019). One of the major limitations to long read sequencing, however, is the higher chances of sequencing errors. In some cases, this may be overcome by increasing the sequencing coverage, and using optimized filtering strategies (Kraft and Kurth, 2019; Mantere et al., 2019). Alternatively, subsequent short read sequencing can be used to optimize for any errors in long read sequencing data (Goodwin et al., 2015).

The time and cost taken to produce results remains a significant limiting factor for most NGS platforms, especially amongst short read sequencing technologies, as library preparations can be time consuming, and batching of samples before processing is required in most cases to reduce the cost of sequencing (Colman et al., 2019; Mayday et al., 2019). Considering the two most popular NGS platforms Illumina and Ion Torrent, both have a range of products that are optimized for speed, cost and amount of sequence data produced (Misyura et al., 2016; Jennings et al., 2017). Illumina provides the lowest cost per base while Ion Torrent generates sequence data faster. In clinical diagnostics and research, these factors would affect the choice of sequencing platform differently, while also providing complementarity. Comparisons between Ion Torrent and Illumina platforms have highlighted Ion Torrents’ suitability for application in clinical diagnostics including automated library preparation, ease of use and speed, however, Illumina offers more accuracy and flexibility (Alekseyev et al., 2018).

While calculating cost, often times the required computational resources both for data storage and bioinformatics analysis required tend to be overlooked. These could be costly especially if NGS strategies like WES or WGS are chosen for studies or tests that require more than one individual. The availability of bioinformatics and computational capacity should also be considered. Therefore, the choice of NGS application for sequencing should take into consideration these various factors, which could have very huge cost implications with little benefit.

## Common Sequencing Errors Associated With NGS Analyses

The nature of errors expected from NGS vary based on the sequencing technology. For instance the common error in Illumina’s sequencing by synthesis technology is single nucleotide substitutions, whilst the Ion Torrent semiconductor sequencing errors mainly come from short deletions, PacBio real-time sequencing errors from CG deletions, and the Life Technologies SOLID technology errors from A-T bias (Voelkerding et al., 2009; Buermans and den Dunnen, 2014; Chimukangara et al., 2017). However, despite the different chances of errors, sequences with a Q30 quality score and above are generally considered reliable, and the number of reads and depth obtained increase the confidence in differentiating the base calls from sequencing errors. Therefore, the ability of NGS platforms to produce vast amounts of sequencing reads, allow for inclusion of only sequence data with high quality, reducing the concern of sequencing errors (Heather and Chain, 2016; Besser et al., 2018). Regardless, it is considered good practice to perform quality control assessments before any analyses.

In order to avoid carrying over sequencing errors into the analysis, a quality control assessment pre-processing step is performed as standard practice. In this step, per-base sequence errors are assessed based on a standard threshold. Sequence reads that do not meet the threshold are removed or trimmed off the erroneous bases if they are found on the flanks. Sequencing artifacts and other contaminants introduced during library preparation such as adapters are trimmed from the sequence reads at this step. Similarly, duplicated reads arising from enrichment bias during sequencing should be removed using tools such as FastQC, Trimmomatic, and fastp.

Analyzing genomes for variation requires correct alignment of sequence reads to reference genomes and accurate variant calling. The alignment step is perhaps the most important step in variant analysis. Inaccurate alignments could lead to incorrect variant calls, therefore, choosing a suitable aligner is crucial (Lindner and Friedel, 2012). Unfortunately, there is no standard method for choosing an aligner leaving the decision to the user who will require a deep understanding of these aligners. To avoid biases due to poor alignment, there are several benchmarking studies comparing the performance of various NGS aligners (Fonseca et al., 2012; Hatem et al., 2013; Shang et al., 2014; Highnam et al., 2015), which could aid in selection of the right aligner. Alternatively, programs like Teaser (Smolka et al., 2015) could be used to assist in the selection of an appropriate aligner and the respective optimum parameters. Errors in alignment are associated with repetitive genomic regions, high genetic diversity between reference genome and target sequences and missing nucleotides or presence of contaminating sequences. It is good practice to assess the quality of the sequence alignments before proceeding with the variant calling step. Tools such as SAMtools provide functionalities to assess the quality of mapped reads based on the PHRED-scaled mapping quality scores (Li et al., 2009). See Pfeifer (2017) for review on generating high quality data for variant analysis.

Variant calling, filtering and annotation is the last step and perhaps the most challenging step in that the outcome is often influenced by factors in previous steps. It is advisable to use one or more variant calling program to increase confidence. The filtering step is necessary to remove false positives caused by sequencing and alignment errors. The choice of filtering program should also reflect the sequencing coverage in order to maximize accuracy. The choice of reference sequence needs to be carefully considered and most importantly, the latest version should be used. Methods used for variant calling have to be highly accurate across millions of base positions in the human genome. It is good practice to always test pipelines whether commercial, open source or in-house pipeline before applying them in any research study or clinical application. Variant calling pipelines can be tested by using a benchmark of high quality genotype datasets. The Genome in a Bottle Consortium (GIAB) is an initiative that has undertaken to analyze and categorize positions in the genome where no confidence calls are likely to be made (Zook et al., 2014). All the methods and reference datasets used by GIAB is freely available at: https://www.nist.gov/programs-projects/genome-bottle. Similarly, the Genetic Testing Reference Materials Coordination Program (GeT-RM) provides appropriately characterized reference materials that could be used for quality control, research, proficiency testing and testing and validation of genotyping pipelines. Reference material provided by GeT-RM include those for testing hereditary genetic disorders among others. This information is also available in the GeT-RM browser hosted in NCBI^33^.

## Analytical, Ethical, and Regulatory Challenges in Analysis of NGS

Whilst NGS has been a fast-growing technology, there remain vast knowledge gaps in the interpretation of NGS data. With several NGS pipelines available, regulating data from NGS still remains challenging, especially when data is to be used for clinical management. This is partly because there is no uniformity in data processing strategies, which results in incomparable and unreproducible data outputs (Gargis et al., 2015; Kanwal et al., 2017; Kulkarni et al., 2018). There are several efforts in place to establish standardized methods of bioinformatics analysis including development of sharable workflows (Baichoo et al., 2018; Kulkarni et al., 2018). The clinical interpretation of identified variants is not standard for all diseases. This issue is being resolved by generating standardized analysis, interpretation and reporting guidelines (Endrullat et al., 2016; Roy et al., 2016; Li et al., 2017; Lindeman et al., 2018; Roy et al., 2018; Hutchins et al., 2019). Incomparable results carry huge implications in clinical applications and should be regulated sooner rather than later (Endrullat et al., 2016).

There are a wide range of ethical issues that obscure acquisition of personal whole genomes or any other genetic data. Careful consideration including genetic counseling on the implications of possible unintended analytical outcomes, must be undertaken before any acquisition of genetic data from patients or clients. Additionally, written consents accompanied by mandatory advice need to be provided. The cost of test needs to be properly addressed, with a strong consideration of insurance authorization, since without insurance, the person or entity is liable for the expenses to be incurred. The issue of secondary findings has to be well relayed to the patient before the test. When confronted by this issue, accredited laboratory guidelines such as those recommended by American College of Medical Genetics and Genomics (ACMG) (Richards et al., 2015; Kalia et al., 2017) or those provided for by credible organizations need to be followed if clinical regulations or local legislation is unavailable (Green et al., 2013).

Handling patient/client genomic data is a sensitive subject entangled in active debate. The regulations safeguarding sharing of personal genomic data for research purposes is of particular concern for many. Governments over the world have introduced legislation to protect the privacy of their citizens’ genomic data. In South Africa, for instance, the Protection of Personal Information Act No. 4 of 2013 (POPIA) was introduced (Staunton et al., 2019). While these regulations have boosted public trust, there are loopholes that still need to be addressed especially when dealing with international collaborations sharing personal genomic data. Case in point, an article appearing in Science magazine (doi: 10.1126/science.aba0343) on Oct. 30, 2019, detailed a scandal where scientists in the famous Wellcome Sanger Institute, United Kingdom, were accused of misusing DNA collected from African people. In contention was a claim that Sanger scientists had developed a commercial chip using the shared DNA which, according to Stellenbosch University and the University of Kwa-Zulu Natal (both who shared 100 DNA samples each) was not part of the material transfer agreements (MTAs). This scandal raised serious ethical questions regarding adherence to MTAs and could jeopardize future genomics research collaborations with the African continent. Additionally, it could erode public trust thereby affecting access to personal genome data.

## Conclusion

Advances in sequencing technology have revolutionized clinical genetic diagnostics and research approaches to identify associated mutations causing Mendelian or complex genetic trait diseases. NGS and 3GS based diagnostic tests for these diseases have been incorporated in clinical medicine. This review discussed the use of family genetic inheritance as an efficient method to identify novel disease-causing mutations using NGS. We also highlighted 3GS platforms that could be used for similar analyses. In addition, we briefly discussed the various bioinformatics tools that are currently available to analyze family based sequencing data. The use of personal genomes for diagnostic or research purposes is not without challenges. These include analytical, ethical and regulatory impediments. We discussed some of the commonly encountered limitations and the remedial efforts that have been put in place and those that still need to be implemented as this fast-developing field of genome sequencing evolves.

## Author Contributions

AMK, VR, and TO conceived and structured the manuscript. AMK, JES, BC, EW, and MF generated the content and wrote the manuscript.

## Conflict of Interest

The authors declare that the research was conducted in the absence of any commercial or financial relationships that could be construed as a potential conflict of interest.

## Footnotes

1 https://www.Illumina.com

2 https://emea.Illumina.com/systems/sequencing-platforms/comparison-tool.html

3 https://www.thermofisher.com

4 https://www.bgi.com

5 https://www.pacb.com

6 https://nanoporetech.com

7 https://gatk.broadinstitute.org/

8 http://novocraft.com/

9 https://www.realtimegenomics.com/news/rtg-core-3-9-rtg-tools-3-9-released

10 https://www.genome.gov/human-genome-project/results

11 https://www.internationalgenome.org/

12 https://gnomad.broadinstitute.org/

13 http://biobank.ctsu.ox.ac.uk/crystal/gsearch.cgi

14 https://www.ncbi.nlm.nih.gov/clinvar/

15 https://www.ncbi.nlm.nih.gov/medgen/

16 https://www.ebi.ac.uk/gwas/

17 http://jjwanglab.org/gwasdb

18 https://www.ebi.ac.uk/eva

19 https://www.humanvariomeproject.org/

20 https://www.clinicalgenome.org/

21 https://www.disgenet.org/

22 https://monarchinitiative.org/

23 http://edgar.biocomp.unibo.it/gene_disease_db/

24 https://www.malacards.org/

25 https://www.orpha.net/

26 https://geno2mp.gs.washington.edu/Geno2MP

27 https://www.omim.org/

28 https://grenada.lumc.nl/LSDB_list/lsdbs

29 http://www.ensembl.org

30 https://genome.ucsc.edu/

31 https://terra.bio/

32 https://www.sevenbridges.com/

33 https://www.ncbi.nlm.nih.gov/variation/tools/get-rm/
